# Is Ultrasonography Useful in the Diagnosis of Nasolabial Cyst?

**DOI:** 10.1155/2014/678541

**Published:** 2014-02-23

**Authors:** Ahmet H. Acar, Ümit Yolcu, Fatih Asutay

**Affiliations:** Department of Oral and Maxillofacial Surgery, Faculty of Dentistry, İnönü University, 44280 Malatya, Turkey

## Abstract

Nasolabial cysts are nonodontogenic cysts that occur beneath the ala nasi. Its pathogenesis is uncertain. Because the nasolabial cyst is a soft tissue lesion, plain radiographs are useless. CT and MRI should be evaluated. In this report, a nasolabial cyst is described including its features on ultrasonography (USG) and CT exams.

## 1. Introduction

Nasolabial cysts are rare nonodontogenic cysts located adjacent to the alveolar process above the apices of incisors [1] and firstly described by Zuckerlandl in 1882 [2]. The term “nasoalveolar cysts” is also used. Clinical properties of nasolabial cysts are usually typical. They can be observed as painless, fluctuating unilateral swellings, at the left alar region. Rarely they can be observed bilaterally. Infection of the cyst may cause pain, and it can drain into the nasal cavity [1]. Because nasolabial cysts are generally not found in the bone tissue, conventional radiographies are not helpful in diagnosis. Instead, other imaging methods such as CT and MRI should be used for diagnosis.

USG is a diagnostic method that is often used for examination of soft tissue lesions and it can also be used in the maxillofacial region. Nasolabial cyst is a lesion of the soft tissue and it can be diagnosed with USG. In this case, CT and USG diagnosis have shown the presence of a 1.5 cm diameter cystic lesion in the soft tissue which has been diagnosed as nasolabial cyst in the histopathologic examination.

## 2. Case Report 

**Figure 1:**
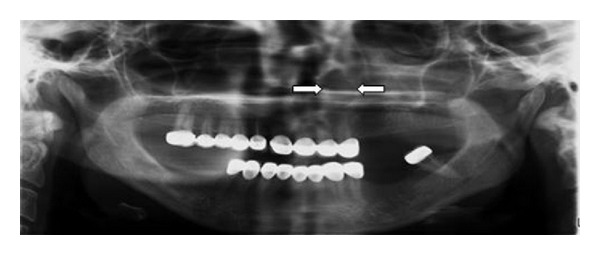
Bone alterations on panoramic radiograph.

**Figure 2:**
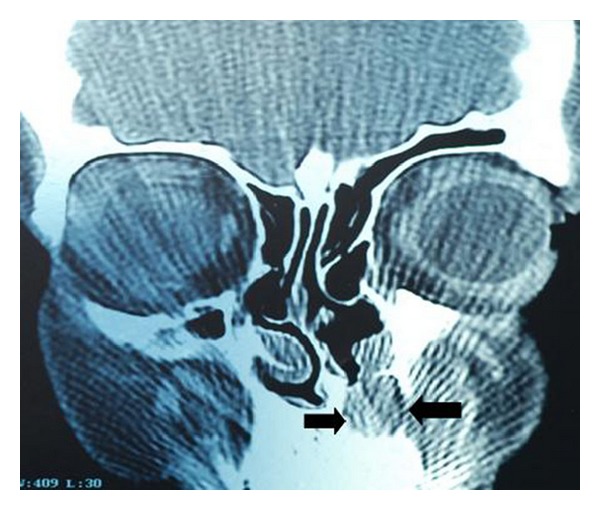
Coronal CT image showed a well-defined 1.5 cm diameter lesion in nasolabial fold.

**Figure 3:**
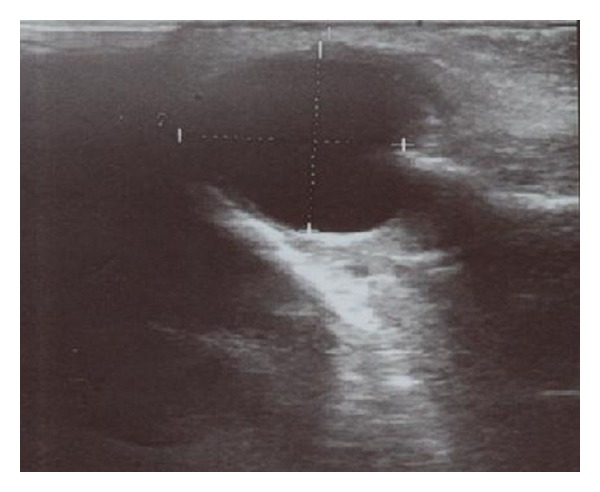
Ultrasonography image showed 1.5 cm diameter soft tissue mass in nasolabial fold.

**Figure 4:**
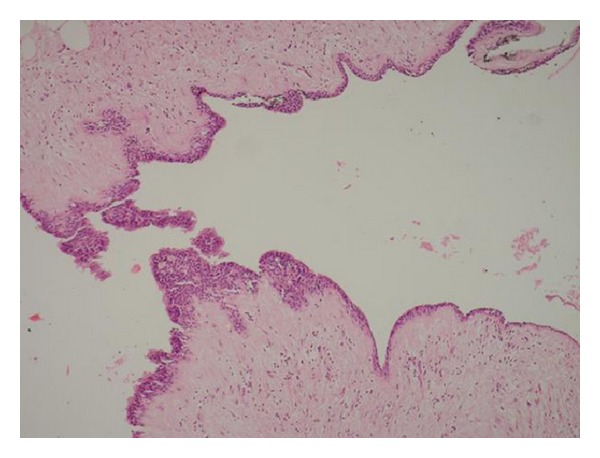
HEx100: microscopic examination of excisional biopsy revealed a cyst, which has an epithelial lining and a fibrous wall. There were three types of epithelial lining in different areas which are cuboidal, stratified squamous, and respiratory type.

A 38-year-old woman presented with a 5-year history of an increasing left nasal ala area swelling. She mentioned difficulty in breathing for about 3 months. She was suffering from pain in nasolabial fold for about 1 year. There was no history of trauma and no medical history. Clinical examination revealed a smooth and fluctuant mass in the left nasal ala area. A floating tumefaction in the nasolabial sulcus was observed. Intraoral examination revealed bulging of the buccoalveolar sulcus by the swelling. There was a facial asymmetry due to bulge on the left side of the nose. The associated teeth tested vital with electrical vitality testing. Nasolabial cysts generally do not cause bone destruction, but in this case bone destruction was observed on panoramic radiograph (Figure 1) and CT image (Figure 2). USG image showed well-defined cystic lesion with 1.5 cm diameter under the skin in left nasolabial fold (Figure 3). After clinical and radiographic examination, the lesion was thought to be a nasolabial cyst. The lesion was removed intraorally under local anaesthesia and it was sent for histopathological examination. According to histopathological examination the lesion was diagnosed as nasolabial cyst. Histopathologic examination has revealed 3 different types of epithelium: cubical, multilayered, and respiratory epithelial cells (Figure 4). After a 2-year follow-up period, the patient had no complaints of pain and swelling. Also the lesion did not recur.

## 3. Discussion

Klestadt was the first person who reported the original theory of nasolabial cyst. According to his theory, the cyst is derived from epithelium trapped in the line of fusion between the lateral nasal and maxillary processes [3]. Now there are two main theories of pathogenesis that have been proposed. Some authors suggest that the cyst arises from nasolacrimal duct epithelium [4]. Others suggest that it is a fissural cyst arising from epithelial remnants entrapped in the developmental fissures between the lateral nasal, globular, and maxillary processes [5]. Amongst all the cysts that are observed in maxilla and mandible, nasolabial cysts have a prevalence of 0.7%. However, due to misdiagnosis this ratio is thought to be higher [6]. Because nasolabial cysts are soft tissue lesion, lesion does not appear in the panoramic radiograph unless bone destruction occurred.

Differential diagnoses of nasolabial cysts should be made with dentoalveolar cysts and incisive foramen cyst. They should also be differentiated from epidermoid or dermoid cysts, unilocular lymphatic malformations, and froncule and dental abscesses [7]. Vitality test results of the teeth that are involved are important [1]. Also, localization of the cyst whether in soft tissue or the hard tissue is important in differentiation. Histopathologic examination is necessary for the definitive diagnosis.

Nasolabial cysts are localised in the soft tissue and they usually are unobserved in conventional radiographies. However, in some cases, if bony destruction is involved, they can be observed radiographically. CT and MRI can be used for diagnosis, localization, and defining the borders of nasolabial cyst. CT is more frequently used in comparison to MRI due to lower costs [8].

USG is another diagnostic method in maxillofacial surgery. USG is used for diagnosis of cellulitis and abscess in the maxillofacial region [9, 10]. Also, it is a helpful imaging method for visualization of lymph node metastasis of oral cancers, for examination of vascular structures, diseases of salivary glands, and in injection biopsy [11, 12]. Akinbami et al. [13] reported that USG is valuable for the differential diagnosis of cysts, tumors, hemangioma, and soft tissue swellings in the cervicofacial region.

USG is inadequate to reflect intrabony defects and lesions and generally used for examination of the soft tissues. It can be safely used in the maxillofacial region to visualize soft tissue lesions like nasolabial cyst. In this case, USG examination of the patient who applied to our clinic displayed a cystic lesion in the soft tissue. Having nonionized character and being inexpensive and more available are some advantages of USG over MRI and CT. In addition to this, the experience of the doctor who performs the USG is an important factor in defining the borders of the lesion accurately. In this case we have shown that USG is useful in the diagnosis of nasolabial cyst. Finally we conclude that direct graphy and USG are adequate in cases in which nasolabial cyst is suspected.
